# Middle meningeal artery embolization for chronic subdural hematoma in octogenarians and nonagenarians: an individual patient pooled meta-analysis

**DOI:** 10.1007/s10143-025-03743-3

**Published:** 2025-08-14

**Authors:** Rahim Abo Kasem, Zachary Hubbard, Julio Isidor, Joshua Venegas, Omar Alwakaa, Felipe Ramirez-Velandia, Muhammed Amir Essibayi, Adnan Rehawi, Christopher S. Ogilvy, Justin H. Granstein, Alejandro M. Spiotta

**Affiliations:** 1 Department of Neurosurgery, Division of Neuroendovascular Surgery, Medical University of South Carolina, Charleston, SC USA; 2 Division of Neurosurgery, Beth Israel Deaconess Medical Center, Harvard Medical School, Boston, MA USA; 3 Department of Neurological Surgery, Montefiore Medical Center, Albert Einstein College of Medicine, Bronx, NY USA; 4 Faculty of Medicine, Damascus University, Damascus, Syria; 5 Medical University of South Carolina, 135 Canon Street, Charleston, SC 29425 USA

**Keywords:** Middle meningeal artery embolization, Nonagenarians, Octogenarians, Chronic subdural hematoma.

## Abstract

Chronic subdural hematoma (cSDH) is a highly common neurosurgical condition with significant burden in the elderly, and patients aged ≥ 80 represent nearly one-third of cases. Middle meningeal artery embolization (MMAE) has emerged as a promising treatment method to reduce hematoma growth and recurrence; however, octogenarians and nonagenarians remain underrepresented in clinical trials. We conducted a systematic review and meta-analysis of individual patient data to evaluate outcomes of MMAE in this high-risk group. We systematically searched PubMed, Scopus, and Web of Science through March 2025 for studies reporting patients aged ≥ 80 years treated with MMAE for cSDH. Individual-level data were extracted and pooled. Clinical, procedural, and radiographic outcomes were summarized. Multivariable mixed-effects logistic regression identified predictors of hematoma resolution and extended hospital stay (LOS ≥ 75th percentile). Twenty-six studies including 86 patients (mean age 83 years; 36% ≥90 years) met inclusion. Most were male (70%) and had comorbidities such as hypertension (57%) and diabetes (21%). MMAE was performed alone in 64% of cases and with surgery in 36%. Transfemoral access was used in 74%, while transradial access (associated with shorter LOS) was underutilized, especially in patients ≥ 90 (3%). Complication and in-hospital mortality rates were low (5.8% and 1.2%, respectively). At discharge, 81% returned home. Complete hematoma resolution occurred in 56%, more likely with adjunctive coiling (aOR 4.01) and less likely with antithrombotic use (aOR 0.12). Extended LOS was associated with age ≥ 90 (aOR 2.1) and general anesthesia (aOR 1.7). MMAE is safe and effective in patients aged ≥ 80. While outcomes are favorable across age groups, transradial access and conscious sedation may reduce hospital stay and anesthesia-related risks in this population. Future trials should prioritize inclusion of this high-risk group to guide age-appropriate, evidence-based care.

**Supplementary Information:**

The online version contains supplementary material available at 10.1007/s10143-025-03743-3.

## Introduction

The burden of chronic subdural hematoma (cSDH) is rising sharply in older adults. Between 2003 and 2016, the incidence of cSDH in the U.S. more than doubled, reaching 58 per 100,000 in the general population and 127 per 100,000 among individuals aged ≥ 80, who represent nearly one-third of affected cases [1]. This trend is driven by increasing life expectancy, widespread neuroimaging, and higher rates of antithrombotic use [2]. As a result, cSDH is projected to become the most commonly treated adult cranial neurosurgical condition by 2030 [3]. Management of these patients, particularly those ≥ 80, poses a challenge due to age-related frailty, medical complexity, and higher perioperative risk, often requiring more nuanced decision-making around anesthesia and procedural invasiveness [1, 2, 4]. 

Traditional surgical approaches, including burr hole drainage and craniotomy, are widely used but carry a significant risk of recurrence, reported in up to 30% of cases [5–7]. Middle meningeal artery embolization (MMAE) has emerged as a minimally invasive technique targeting the pathophysiology of cSDH through devascularization of neomembranes [5]. Favorable outcomes have been reported in patients unable to stop antithrombotic therapy or with recurrent hematomas, but those ≥ 80 years remain underrepresented in clinical trials evaluating MMAE [6]. Given the demographic shift and the increasing need for less invasive yet effective therapies, defining the safety and efficacy of MMAE in the octogenarians and nonagenarians is critical for evidence-based, age-appropriate treatment guidelines. We conducted a systematic review and individual patient data meta-analysis to evaluate middle meningeal artery embolization (MMAE) in adults aged ≥ 80 with chronic subdural hematoma (cSDH). The objectives were to characterize clinical, procedural, and radiographic outcomes within this high-risk population; to identify predictors of hematoma resolution and extended hospital stay; and to evaluate the impact of access route and anesthesia strategy on postoperative recovery.

## Methods

### Study design and registration

This study is a systematic review and meta-analysis of individual patient data (IPD) that was conducted following the Preferred Reporting Items for Systematic Reviews and Meta-Analyses (PRISMA) guidelines [7]. 

### Systematic literature search strategy

A systematic search of the literature was performed using PubMed, Scopus, and Web of Science databases for relevant studies published up to March 31, 2025. We employed a comprehensive search strategy combining Medical Subject Headings (MeSH) terms and keywords related to (“chronic subdural hematoma” OR “subdural hematoma” OR “CSDH” OR hematoma) AND (“middle meningeal artery embolization” OR MMEA OR MMAE OR “embolization”). The specific search syntax was adapted for each database. We used a syntax without elderly filters to include papers reporting outcomes across different age groups. A manual search of the references in included studies was also conducted to identify any potentially missed studies.

### Study selection

Two independent reviewers screened titles and abstracts yielded by the search strategy. Potentially relevant articles underwent full-text review against pre-defined inclusion and exclusion criteria. Discrepancies between reviewers were resolved through discussion or consultation with a third reviewer.

- Inclusion Criteria:
  - Studies reporting patients diagnosed with cSDH who are ≥ 80 years old and treated with MMAE.
  - Included study designs were case reports, case series, retrospective or prospective cohort studies, and observational studies.
  - Studies providing sufficient data to evaluate clinical presentation, management, and outcomes.
  - Studies published in the English language containing at least one relevant case.
- Exclusion Criteria:
  - Studies with inadequate or incomplete information.
  - Studies not presenting data specifically on ≥ 80 years old cSDH patients.
  - Publications that were not original research articles (e.g., systematic reviews, meta-analyses, conference abstracts without full text, reviews, editorials, expert opinions) or were not in English.

### Data extraction

Data from the included studies were extracted independently by two reviewers using a standardized data collection form. For each identified patient, the following variables were collected where available: demographic data such as, age and sex, alongside baseline comorbidities including hypertension, diabetes mellitus, atrial fibrillation, coronary artery disease, other significant cardiac conditions, liver disease, chronic kidney failure, history of or concomitant malignancy, and prior stroke/TIA. Clinical presentation details were recorded, noting pre-procedural use of antiplatelets or anticoagulants, whether the patient was asymptomatic or presented with symptoms like focal weakness, headache, altered mental status, or gait instability/falls/limb weakness, and if concurrent surgical decompression was performed. Characteristics of the subdural hematoma itself, specifically its laterality (left, right, or bilateral) and suspected cause (spontaneous or traumatic), were also documented. Furthermore, specifics of the MMAE procedure were collected, covering the vascular access approach (transradial [TRA] or transfemoral [TFA]), type of anesthesia used (general anesthesia [GA], conscious sedation [CS], or monitored anesthesia care), and the embolic agents and specific materials employed (e.g., PVA particles, Onyx, nBCA glue, SQUID, coils, or combinations). If individual data were not available, data were simulated based on mean and error, reported proportions, or numerical range.

### Outcome measures

Clinical outcomes and follow-up data were gathered, including length of hospital stay, discharge destination (home vs. care facility), in-hospital mortality, and any complications. readmission or retreatment events, duration of follow-up time, radiological status of the SDH, and midline shift compared to presentation (categorized as complete resolution, improvement, stability, or worsening). Extended LOS (eLOS) was defined as the upper quartile (≥ 75th) of the median duration of hospital stay.

### Statistical analysis

Descriptive statistics were generated to characterize the pooled individual patient data (IPD) obtained through the systematic review. The distribution of continuous variables was assessed (e.g., via visual inspection of histograms and Shapiro-Wilk tests), and variables were subsequently summarized as mean ± standard deviation (SD) or median [interquartile range (IQR)] as appropriate. Categorical variables were presented as frequency counts and percentages (n, %).

Inter-study heterogeneity was formally assessed using Cochrane’s Q statistic and quantified using the I² index [8]. 

For the meta-analysis of predictors, multivariable mixed-effects logistic regression models were developed using the pooled IPD. This approach was chosen to identify potential predictors of outcomes while accounting for potential clustering effects and baseline outcome variations between studies, typically by incorporating random intercepts for each included study. Variable selection for the final model was performed using a stepwise backward elimination procedure, commencing with a model including all candidate predictors deemed clinically relevant or statistically plausible. We used a significance cutoff of *p* < 0.1 for variable inclusion in the multivariable model, which is standard in regression analyses with limited sample sizes to avoid excluding potentially important predictors. The calibration and goodness-of-fit of the final multivariable model were evaluated using the Hosmer-Lemeshow test.

Across all analyses, a two-sided p-value less than 0.05 was considered statistically significant. All statistical analyses were conducted using R version 4.4.3 [9]. Specific R packages utilized for data manipulation, analysis, and visualization included ‘dplyr’, ‘tidyr’, ‘forcats’, ‘ggplot2’, ‘forestplot’, ‘patchwork’, and ‘ggrepel’.

## Results

### Study selection

**Fig. 1:**
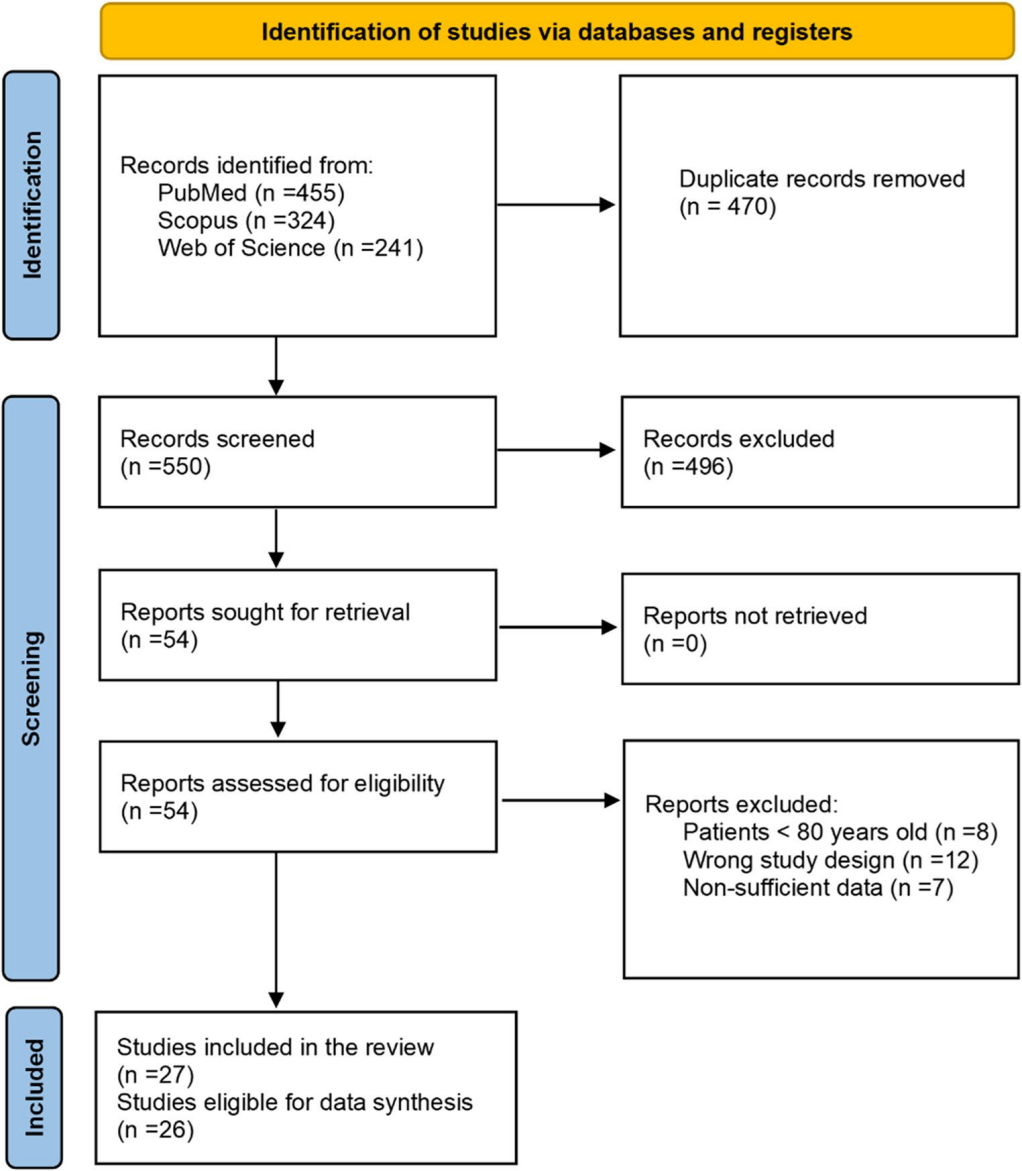
Flow diagram of literature review and study selection process

Our search key retrieved a total of 550 studies, of which 54 were included based on title and abstract screening. Based on a full-text review, we identified 26 manuscripts with a pooled total of 86 patients aged 80 years or older who underwent MMAE ± surgical evacuation for cSDH (Fig. 1, Supplemental Materials, Table 1).

### Patient population and comorbidities

**Fig. 2:**
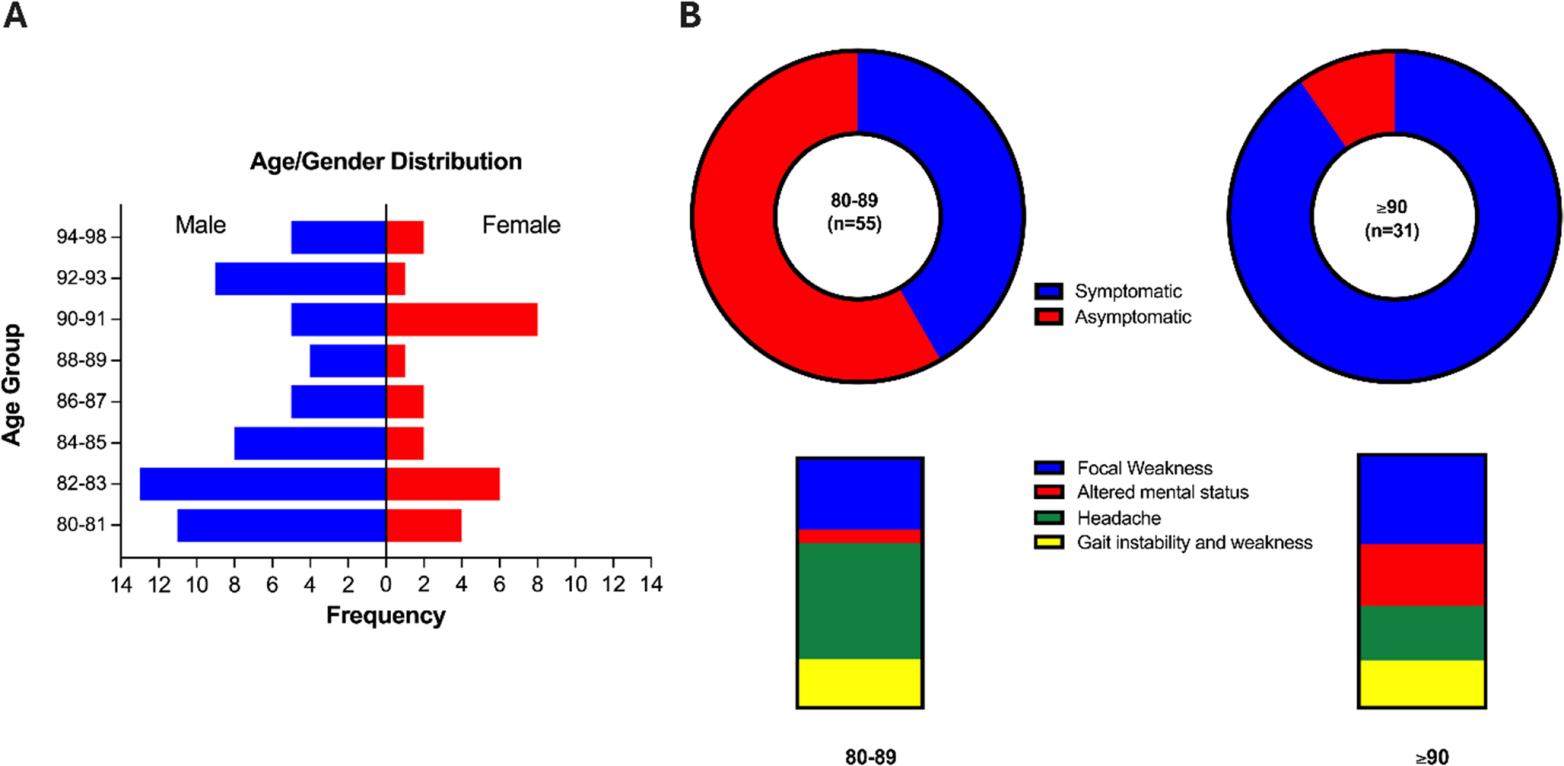
Demographics and presenting characteristics of patients with chronic subdural hematoma (cSDH), stratified by age groups 80–89 and ≥ 90 years. **A** Age and sex distribution, **B** Symptomatology and distribution of presenting symptoms

This study evaluated MMAE for the treatment of cSDH in 86 patients aged 80 years or older. Males accounted for the majority of patients (69.8%, *n* = 60). **(**Table 1; Fig. 2A) The cohort was divided into two main age groups: 64% (*n* = 55) were aged 80–89 years, and 36% (*n* = 31) were 90 years or older. Patients aged 90 or above were more likely to be symptomatic (90%) compared to those aged 80–89 (64%). **(**Table 1; Fig. 2B) Hypertension was reported in 57% (*n* = 49) of patients, followed by diabetes mellitus (DM) (20.9%, *n* = 18), and a history of malignancy (16.3%, *n* = 14). Coagulopathy (14.0%, *n* = 12), prior stroke/TIA (12.8%, *n* = 11), and coronary artery disease (12.8%, *n* = 11) were less common (Table 1).

**Table 1:** Baseline demographic, clinical, and radiographic characteristics of the study cohort

Baseline Characteristics	Total (86)
Sex, Females, n (%)	26 (30.2)
Age	83 [82–87]
Age groups, n (%)	
80–89	55 (64.0)
90 or above	31 (36.0)
Comorbidities, n (%)	
Hypertension	49 (57.0)
Diabetes Mellitus	18 (20.9)
Coronary heart disease	11 (12.8)
Atrial Fibrillation	9 (10.5)
Congestive Heart Failure	1 (1.2)
Heart valve replacement	1 (1.2)
Liver disease	3 (3.5)
Chronic kidney disease (CKD)	5 (5.8)
History of Concomitant malignancy	14 (16.3)
Prior stroke/Transient Ischemic Attack	11 (12.8)
Venous sinus thrombosis	1 (1.2)
Iatrogenic Dural Arteriovenous Fistula	1 (1.2)
Cranial Trauma	25 (29.1)
Coagulopathy	12 (14.0)
Antiplatelets, *n* (%)	27 (31.4)
Anticoagulants, *n* (%)	5 (5.8)
Asymptomatic, *n* (%)	23 (26.7)
Focal weakness, *n* (%)	29 (33.7)
Headache, *n* (%)	30 (34.9)
Altered mental status, *n* (%)	12 (14.0)
Gait instability/falls/Limb weakness, *n* (%)	18 (20.9)
**Laterality**, n (%)	
Left	42 (48.8)
Right	31 (36.0)
Bilateral	13 (15.1)
**Etiology**, n (%)	
Spontaneous	61 (70.9)
Traumatic	25 (29.1)

### Etiology and clinical presentation

The spontaneous etiology of cSDH was reported in 70.9% of cases (*n* = 61), and 29.1% (*n* = 25) were attributed to trauma. Pre-existing coagulopathy was reported in 14.0% (*n* = 12) of the cohort. Furthermore, iatrogenic risk from antithrombotic therapy was reported, with 31.4% (*n* = 27) of patients on antiplatelets and 5.8% (*n* = 5) on anticoagulants **(**Table 1) The cSDH were asymptomatic in 26.7% (*n* = 23) patients. In symptomatic cSDH, headache (34.9%, *n* = 30) and focal weakness (33.7%, *n* = 29) were the most common presenting symptoms (Table 1). However, the symptom distribution varied by age group; compared to the 80–89 group, patients aged 90 or older presented relatively more often with gait instability/limb weakness and with altered mental status (Fig. 2B). The cSDH occurred predominantly unilaterally, affecting the left side in 48.8% (*n* = 42) and the right side in 36.0% (*n* = 31), with 15.1% (*n* = 13) being bilateral (Table 1).

### Procedural characteristics

**Fig. 3:**
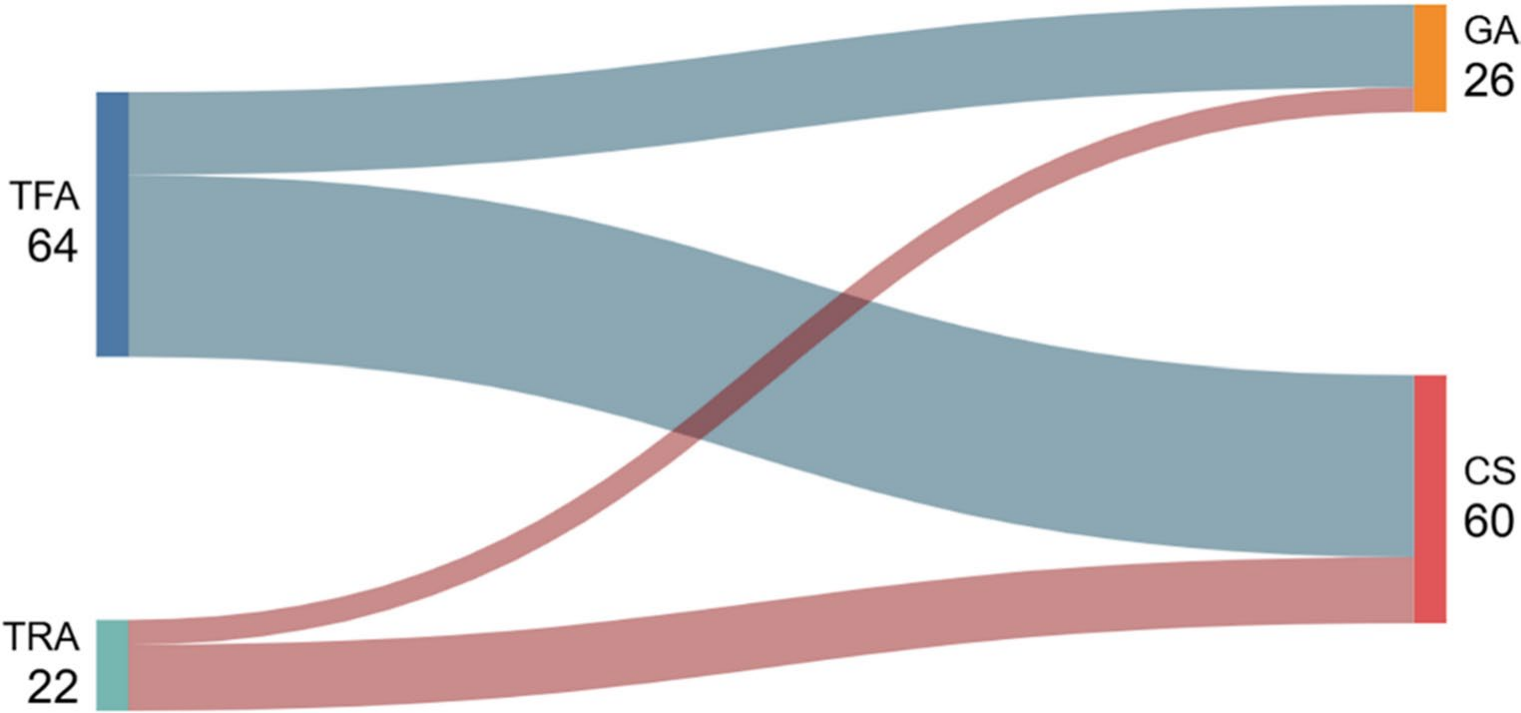
Sankey diagrams showing the distribution between vascular access route; Transfemoral (TFA) and Transradial (TRA), and anesthesia type, General anesthesia (GA) and Conscious sedation (CS), in **octogenarians and nonagenarian** undergoing MMAE

TFA was utilized in 74.4% (*n* = 64) of cases, and TRA was employed in 25.6% (*n* = 22). The use of TRA varied by age group, with 38.2% (*n* = 21) in the 80–89 age group compared to 3% (*n* = 1) in patients aged 90 or older. Conscious sedation was used in 69.8% (*n* = 60) of cases, and general anesthesia was administered in 30.2% (*n* = 26). Conscious sedation usage differed by age, noted in 69% (*n* = 38) of patients aged 80–89 and 79.9% (*n* = 22) of those aged 90 or older (Table 2; Fig. 3, Supplemental Materials, Fig. 1).

**Table 2:** Procedural characteristics and embolization details of MMAE cases

Procedural details	Total (86)
MMAE alone	55 (64.0)
MMAE + surgical evacuation	31 (36.0)
MMAE + Burr hole	26 (30.2)
MMAE + Craniotomy	5 (5.8)
**Access**	
Transradial (TRA)	22 (25.6)
Transfemoral (TFA)	64 (74.4)
**Anesthesia**	
General anesthesia (GA)	26 (30.2)
Conscious sedation (CS)	60 (69.8)
**Embolic agents**	
Particles	25 (29.1)
Liquids	33 (38.4)
Onyx	1 (1.2)
nBCA glue	25 (29.1)
SQUID-18	7 (8.1)
Coils	7 (8.1)
Particles with coils	12 (14.0)
nBCA glue with coils	8 (9.3)
Particles with nBCA	1 (1.2)

## Embolic agents and treatment strategy

**Fig. 4:**
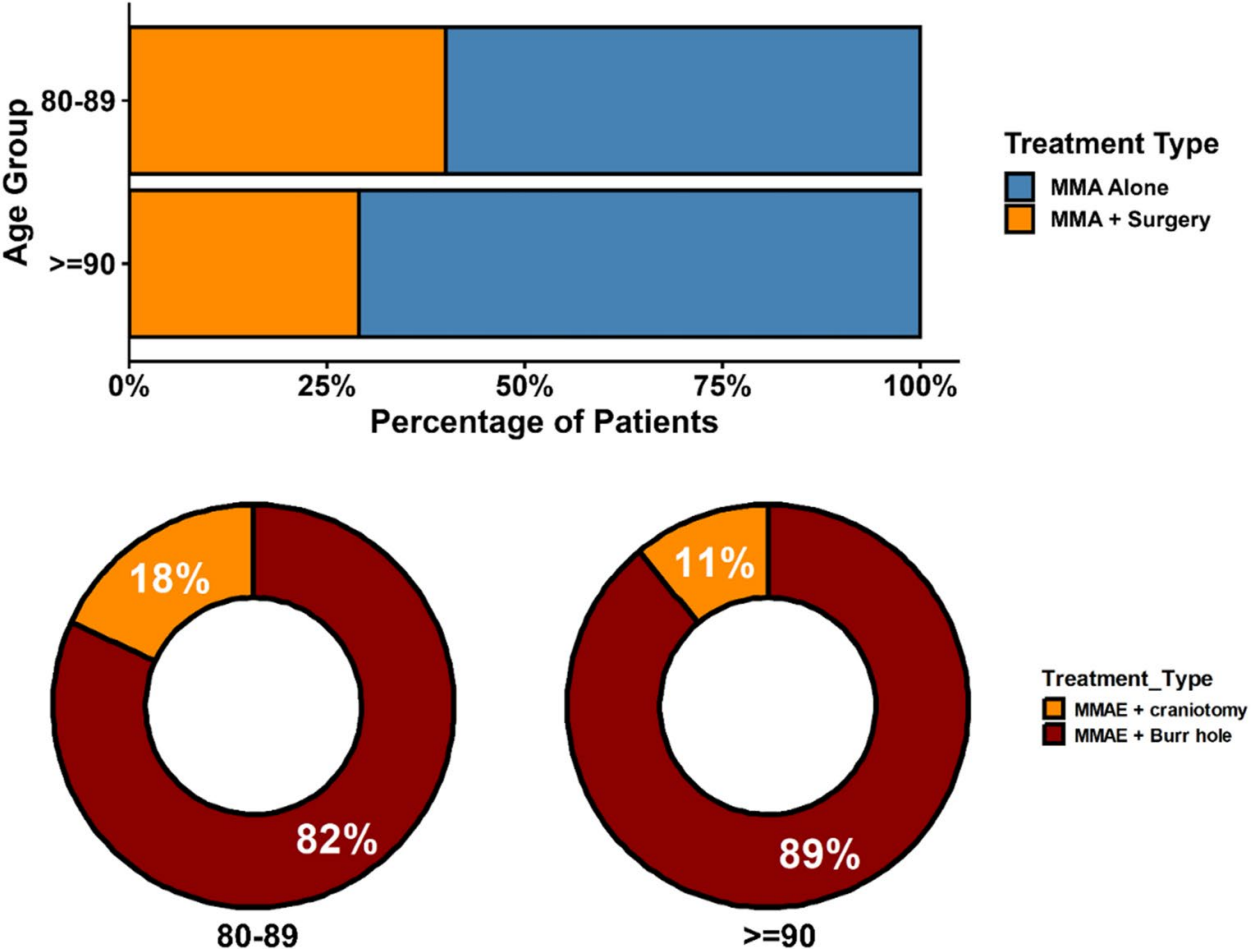
Treatment approach: MMAE alone vs. MMAE with surgical evacuation (Burr hole or carniotomy) of patients with chronic subdural hematoma (cSDH), stratified by age groups 80–89 and ≥ 90 years

A variety of embolic agents were utilized, with liquid agents (including Onyx, nBCA glue, SQUID; 38.4%, *n* = 33) and particles (29.1%, *n* = 25) being the most common single agents, alongside various combinations primarily involving coils (Table 2). Regarding the overall treatment strategy, MMAE alone was performed in 64.0% (*n* = 55) of cases. The remaining 36.0% (*n* = 31) were managed with a hybrid strategy involving both MMAE and surgical evacuation. In these cases, the surgical procedure was burr hole drainage for 30.2% (*n* = 26) of the total cohort and craniotomy for 5.8% (*n* = 5) (Table 2; Fig. 4).

## Clinical outcomes

**Fig. 5:**
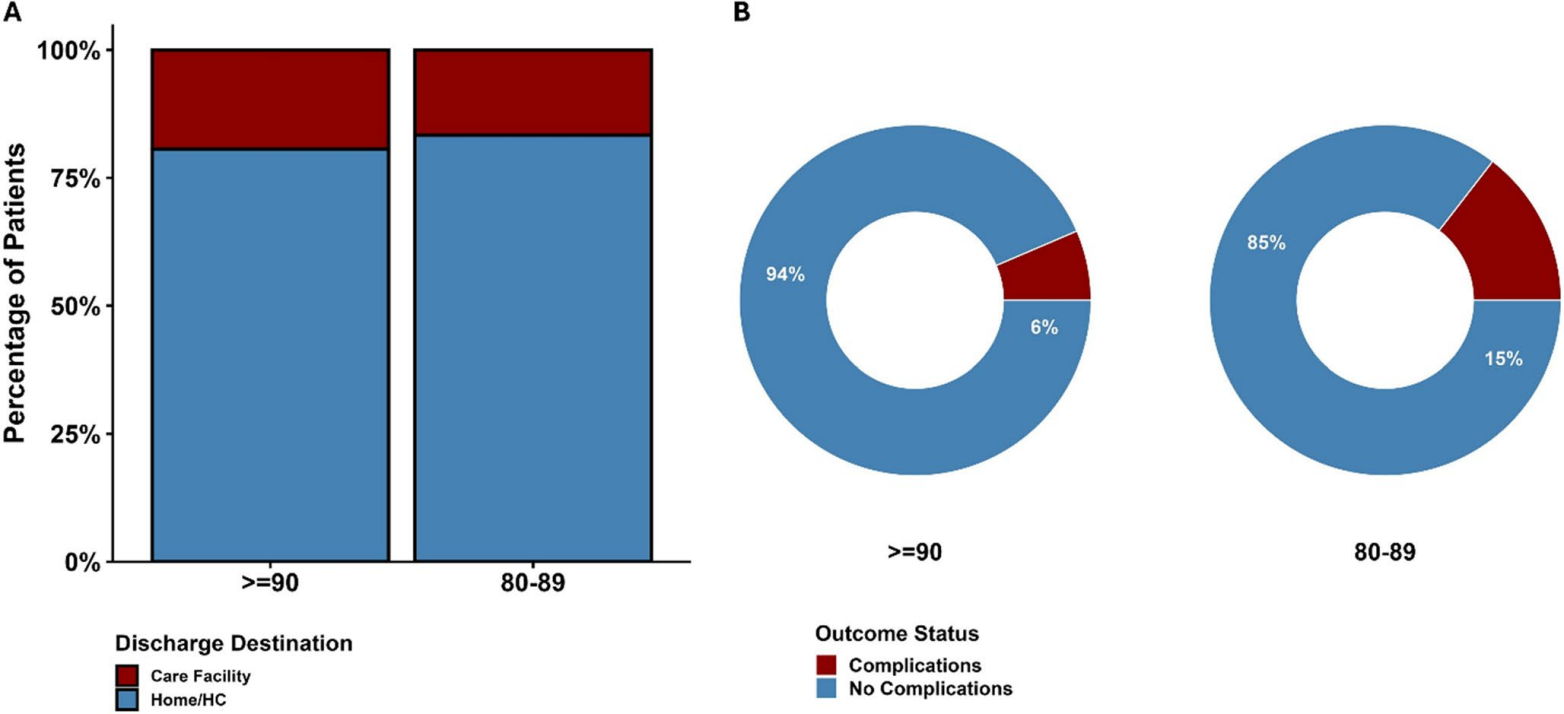
Clinical outcomes stratified by age group in patients undergoing MMAE for cSDH. **A** Discharge destination (home vs. care facility). **B** Procedural complication rates by age group

Procedural complications occurred in 5 patients (5.8%): neurological deterioration (1 case), facial nerve palsy (1 case), dural arteriovenous fistula (2 cases), and femoral artery pseudoaneurysm (1 case). One patient (1.2%) died during hospitalization. The median follow-up was 5 months (IQR: 3–6 months), with 3 patients requiring readmission or retreatment. Complication rates by age group were 6.5% (2 cases) for patients aged ≥ 90 and 10.9% (6 cases) for those aged 80–89 (Table 3; Fig. 5B). Upon discharge, 70 patients (81.4%) returned home, 15 (17.4%) required assisted living; no difference between age groups (Table 3; Fig. 5A).

**Table 3:** Clinical, radiographic, and disposition outcomes following MMAE

Outcomes	Total (86)
Complications of MMAE, *n* (%)	5 (5.8)
Neurological deterioration	1 (1.2)
Facial nerve palsy	1 (1.2)
Dural arteriovenous fistula	2 (2.4)
Femoral artery pseudoaneurysm	1 (1.2)
**Length of stay**,** median (IQR)**	5 [3–7]
Discharge Destination, *n* (%)	
Home	70 (81.4)
Assisted living facility	15 (17.4)
In-hospital mortality	1 (1.2)
Time to last FU, median (IQR), months	5 [3–6]
Readmission/Retreatment, *n* (%)	3 (3.5)
SDH status compared to presentation, *n* (%)	
Complete resolution	48 (55.8)
Improved, including persistent full resolution on previous imaging	24 (27.9)
Stable/no change if not fully resolved	11 (12.8)
Worsened including death	3 (3.5)
Evidence of midline shift, *n* (%)	20 (23.3)

**Fig. 6:**
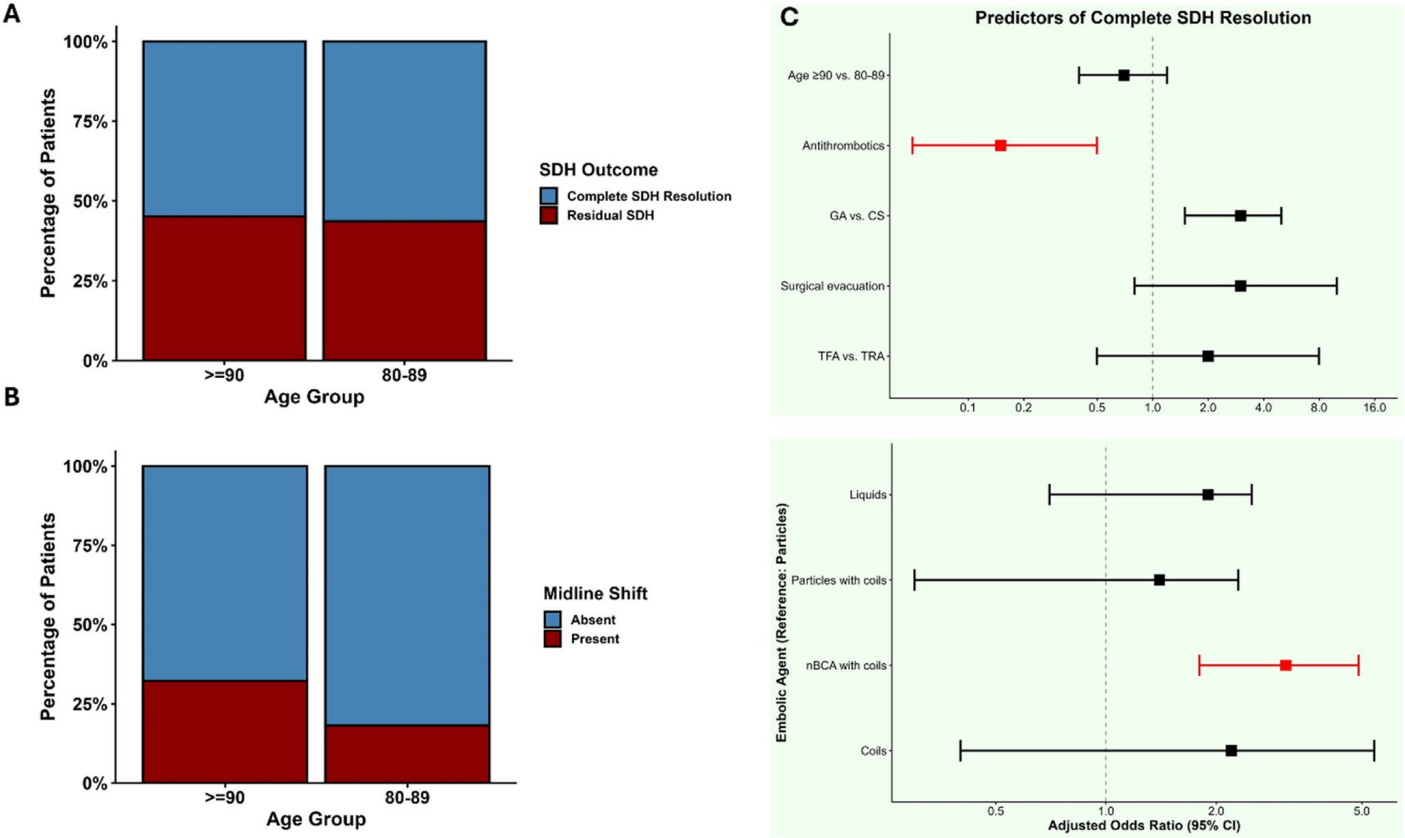
Clinical outcomes and predictors stratified by age group in patients undergoing MMAE for cSDH (**A**) Subdural hematoma (SDH) resolution status (**B**) Presence of midline shift on imaging (**C**) Forest plot of multivariable predictors of complete SDH resolution. **Abbreviations:** CS = conscious sedation; GA = general anesthesia; SDH = subdural hematoma; TFA = transfemoral access; TRA = transradial access; aOR = adjusted odds ratio; CI = confidence interval

Radiologically, SDH completely resolved in 48 patients (55.8%), improved without full resolution in 24 (27.9%), was stable without improvement in 11 (12.8%), and worsened or resulted in death in 3 (3.5%). Antithrombotic use was associated with decreased odds of complete resolution (aOR 0.14, 95% CI: 0.001–0.48), while MMAE with combined nBCA and coils was associated with increased odds of complete resolution (aOR 3.3, 95% CI: 1.8–5) (Table 3; Fig. 6A, C). Midline shift was present in 20 patients (23.3%), occurring more frequently in patients aged 90 or older (32.3%, *n* = 10) compared to those aged 80–89 (18.2%, *n* = 10) (Table 3; Fig. 6B).

**Fig. 7:**
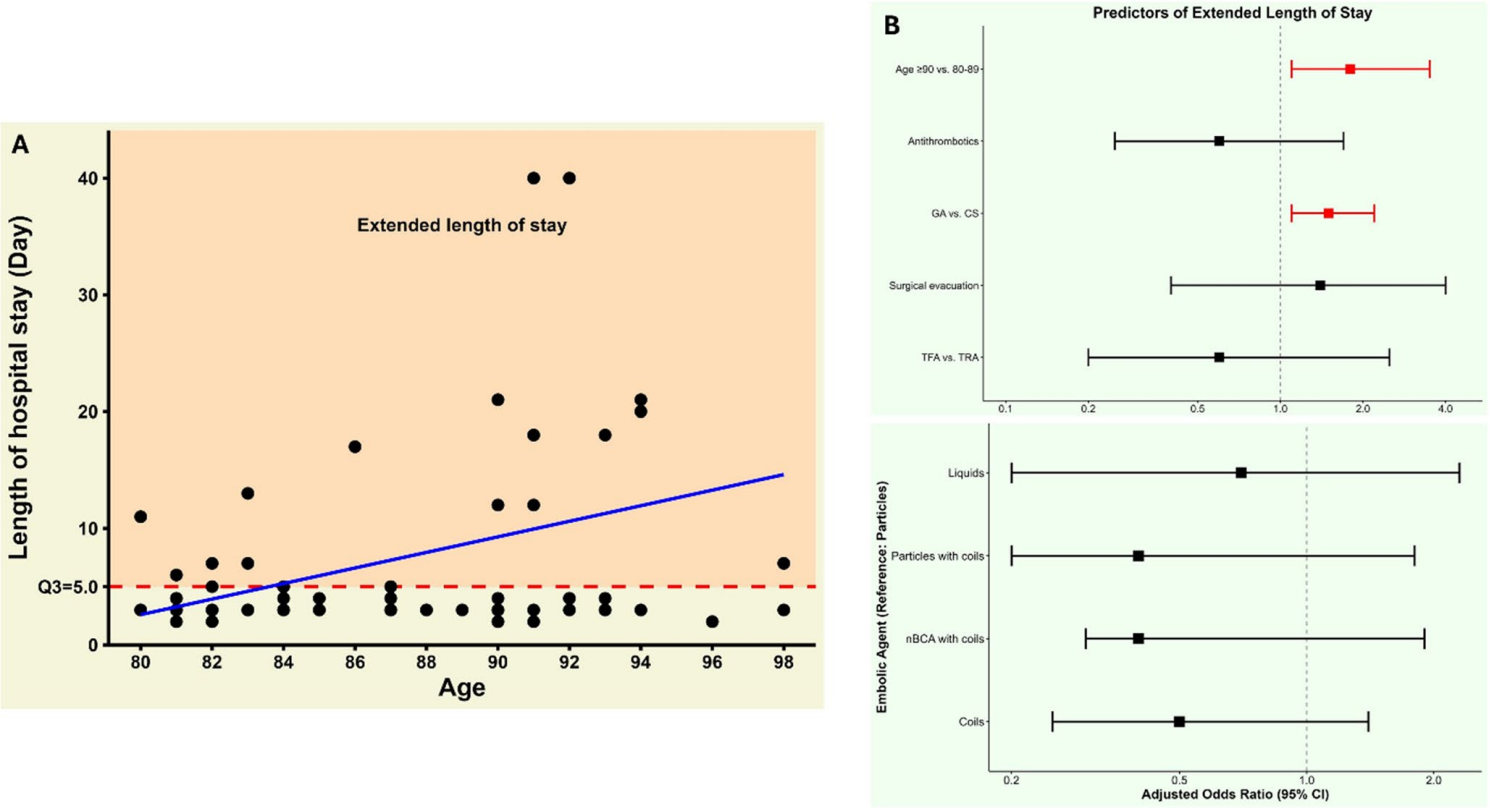
Relationship between age and length of hospital stay, **A** with the upper quartile threshold (Q3 = 5 days) indicating extended stay. **B** Forest plot of multivariable predictors of extended length of hospital stays. **Abbreviations:** CS = conscious sedation; GA = general anesthesia; SDH = subdural hematoma; TFA = transfemoral access; TRA = transradial access; aOR = adjusted odds ratio; CI = confidence interval

Median hospital length of stay was 5 days (IQR: 3–7), with extended length of stay (> 5 days) predicted by age 90 or older (aOR 1.9, 95% CI: 1.2–3.7) and general anesthesia (aOR 1.7, 95% CI: 1.2–2.3) (Table 3; Fig. 7A, B).

## Discussion

This study presents the first pooled analysis of patients aged 80 or older undergoing MMAE for cSDH. Despite cSDH affecting the elderly and the rapid expansion of MMAE usage, this demographic remains underrepresented in literature, with only 86 patients identified in published real-world studies. MMAE demonstrated a favorable safety profile, showing low complication (5.8%) and in-hospital mortality (1.2%) rates, with most patients (81%) discharged home. Combined nBCA with coiling during MMAE correlated with higher odds of complete hematoma resolution, while antithrombotic use reduced those odds. Transradial access was used less frequently in patients > 90. In patients aged ≥ 90, rates of symptomatic collections were higher, the degree of midline shift was greater, and were often treated under conscious sedation via TFA. While radiographic outcomes were similar across age groups, patients aged 90 years or older faced a higher risk of prolonged hospitalization.

Accumulating histologic and anatomic evidence suggests that the fragility of neovasculature within the dural border cell layer plays a central role in cSDH. Damage to these capillaries leads to leakage of blood products into the potential space between the inner and middle dural layers, initiating a cyclical inflammatory and angiogenic response [10]. This paradigm shift from viewing cSDH as a passive venous bleed to an active, self-perpetuating inflammatory process has reshaped treatment approaches, particularly in older patients with cerebral atrophy or minor trauma [10–12]. Recent results from the EMBOLISE [13], STEM [14], and MEMBRANE [15] trials now provide Level 1 evidence supporting middle meningeal artery embolization (MMAE) as an effective adjunct in the management of chronic subdural hematoma (cSDH). While the MAGIC-MT [16] trial did not meet its primary efficacy endpoint, it did demonstrate a favorable safety profile with fewer serious adverse events in the MMAE arm. As the evidence base grows and MMAE is increasingly adopted in real-world practice, it remains critical to identify patient subgroups who may derive the greatest benefit, as well as those who may not respond favorably to embolization.

**Fig. 8:**
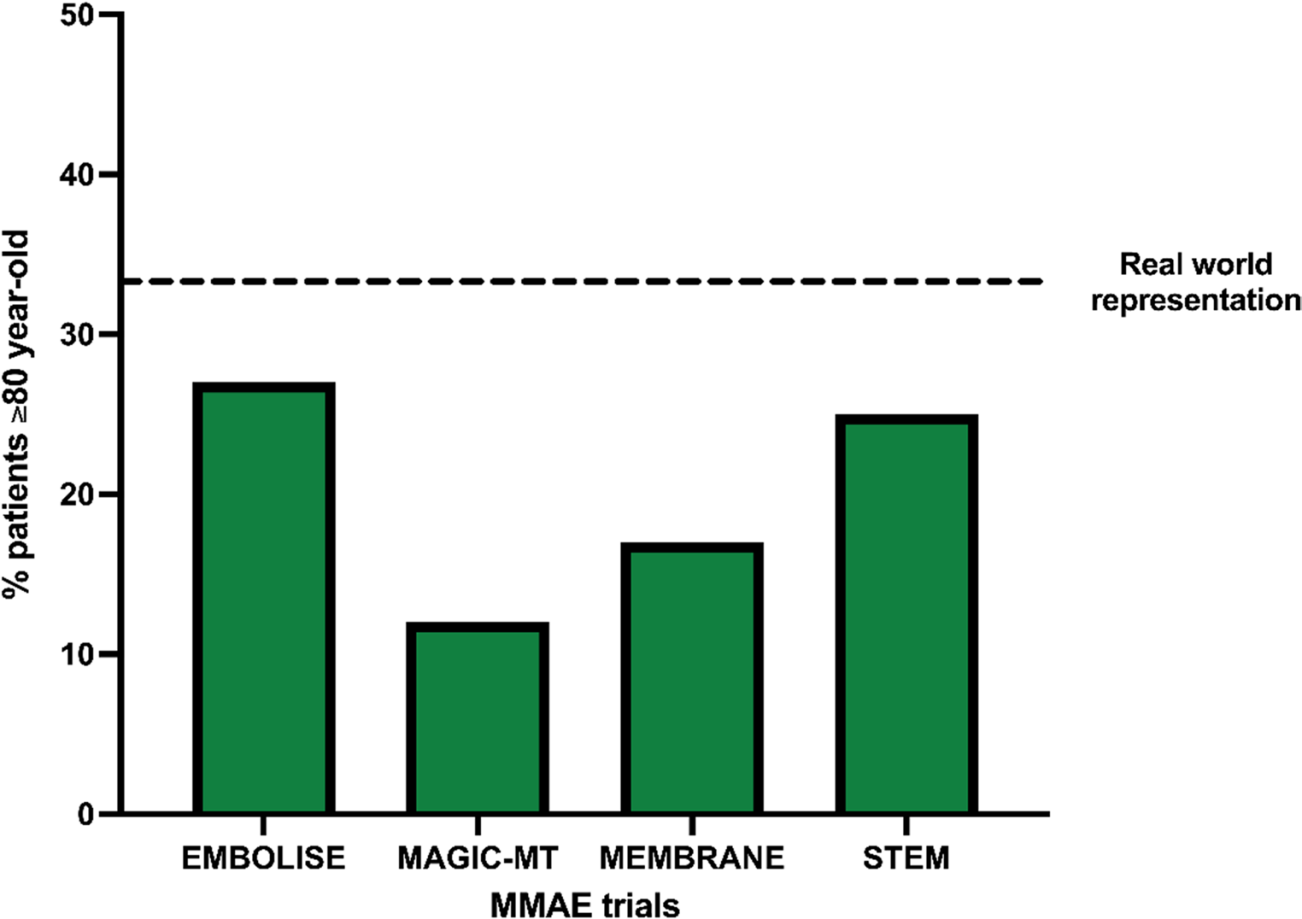
Proportion of patients aged ≥ 80 years estimated using reported means and standard deviations in the MMAE arms, assuming a normal distribution. Dashed line indicates real-world benchmark where ≥ 80-year-olds account for > 33% of cSDH cases

Patients aged 80 years or older remain significantly underrepresented in the trials on MMAE for cSDH. Patients over 90 are sometimes intrinsically excluded, and subgroup outcomes have not been reported. Across trials, mean ages in the MMAE arms ranged from 67.5 to 73 years (SD ~ 10.4–11.0). (Table 4) Assuming a normal distribution, only an estimated 11–27% of participants were ≥ 80 years old, below their representation in real-world cSDH populations, where individuals ≥ 80 account for nearly one-third of all cases. (Fig.8) This discrepancy highlights a key sampling bias and limits the generalizability of current trial findings to those aged ≥ 80 years, who bear the greatest disease burden yet remain the least studied. MMAE offers a compelling therapeutic profile for patients aged ≥ 80 years, who often present with higher anesthetic and surgical risk [17–19]. Unlike traditional operative evacuation, MMAE can be performed under local or moderate sedation, significantly reducing the physiological burden of general anesthesia, an important advantage in a population prone to cardiopulmonary complications, delirium, and postoperative deconditioning. Conscious sedation facilitates procedural safety while maintaining hemodynamic stability, even in patients with significant comorbidities [20, 21]. However, in our experience, one notable drawback of conscious sedation is the risk of patient motion during the procedure, particularly when embolic agents with meningeal irritating properties are used. Emerging strategies, such as pre-treating the MMA with analgesic agents prior to embolic administration, may help minimize discomfort and mitigate patient movement, although this approach remains under investigation. While the TRA was used less frequently in this age group, possibly due to anatomical challenges, vessel tortuosity, calcification or operator familiarity, it confers additional benefits, including enhanced procedural tolerance, earlier mobilization, and reduced length of stay [22–24]. Nonetheless, definitive conclusions regarding the optimal access route (TRA vs. TFA) or choice of anesthesia must be individualized based on patient-specific anatomical and clinical variables, highlighting the need for further research to guide treatment decision-making.

**Table 4:** Age representation in randomized controlled trials of MMAE for cSDH

	EMBOLISE	MAGIC-MT	MEMBRANE	STEM
Treatment groups	MMAE + surgery/Surgery alone	MMAE + surgery and MMAE alone/Surgery alone and conservative	MMAE + surgery/Surgery alone and conservative	MMAE + surgery and MMAE alone/Surgery alone and conservative
No. of patients	600	722	376	310
Age in MMAE arm	73.0 ± 11.0	67.5 ± 10.4	70 ± 10.4	72.8 ± 10.4

Antithrombotic use was associated with significantly decreased odds of complete hematoma resolution after MMAE in our cohort of patients aged ≥ 80. This observation is consistent with large multicenter studies in broader age groups and may be attributed to the fact that subdural collections resolve through gradual thrombosis over time [25]. Additionally, the elderly have higher vascular fragility and are more prone to rebleeding, which may may lead to re-accumulation of the collection. As such, optimizing peri-procedural antithrombotic management and carefully weighing the risks and benefits of therapy are essential.

Our pooled meta-analysis found that procedural complications after MMAE in patients aged 80 years or older remained low, with only 5.8% experiencing any complication and a 1.2% in-hospital mortality. Importantly, most patients returned home after the procedure. These findings align with prior meta-analyses and systematic reviews reporting pooled complication rates for MMAE in the 3–5% range, comparable to conventional surgery for cSDH [26]. The observed complications, including neurological deterioration, cranial nerve injury, vascular events, and rare access site complications, mirror those previously reported. Notably, severe complications such as stroke and blindness were exceedingly uncommon, and most adverse events reflected either procedural factors or the high baseline comorbidity burden typical of this population.

Recent multicenter studies have reported no significant differences in efficacy or safety among various embolic agents used for MMAE in chronic subdural hematoma [27]. However, our pooled analysis of patients aged 80 and older indicates that combining nBCA with coils is associated with significantly higher odds of complete hematoma resolution. This may be due to the complementary effects of each material: coils achieve proximal arterial occlusion, while nBCA enables deeper penetration into the subdural neomembranes, targeting the underlying neovascular supply. Given the ongoing debate and lack of randomized data on material combinations, further research is needed.

Although randomized trials support MMAE for cSDH, patients aged ≥ 80 have been consistently underrepresented, and those ≥ 90 were explicitly excluded, despite both groups bearing the highest disease burden. This highlights the need for higher-level evidence beyond the scope of this study. Nonetheless, our study offers the first pooled analysis centered on octogenarians and nonagenarians undergoing MMAE, drawing from a growing body of high-interest literature. It provides Level 3 evidence to inform treatment decisions [28] and emphasizes the need for future trials to prioritize appropriate age representation. This study is limited by its reliance on published reports, introducing risks of publication bias, selective reporting, and overlapping cohorts (full-text articles were reviewed to exclude duplicate populations). However, a key limitation of the current literature is the lack of granular data on factors such as laterality of the target vessel, aortic arch type, hematoma size, symptom severity, and rationale for selecting anesthesia or embolic agents, which are factors that can influence treatment outcomes. We recommend that future prospective studies systematically include these variables.

## Conclusion

MMAE, particularly when performed via transradial access and under conscious sedation, may offer significant procedural advantages in octogenarians and nonagenarians by minimizing the risks associated with general anesthesia while achieving comparable radiographic outcomes. Future studies should specifically assess the feasibility and safety of the transradial approach in patients aged ≥ 80. Additionally, randomized trials must ensure that octogenarians and nonagenarians are represented in proportions reflective of their real-world prevalence in the cSDH population to enhance external validity and guide evidence-based treatment decisions for this high-risk group.

## Supplementary Information

Below is the link to the electronic supplementary material.

Supplementary Material 1 (DOCX 188 KB)

## Data Availability

Data supporting the findings of this study are included in the manuscript and its supplementary materials. Additional details are available from the corresponding author upon reasonable request.

## Declarations

### Clinical trial registration

This manuscript is a systematic review and individual patient pooled meta-analysis, not a clinical trial. Therefore, clinical trial registration is not applicable.
