# Supplementary material for: Middle meningeal artery embolization for chronic subdural hematoma in octogenarians and nonagenarians: an individual patient pooled meta-analysis
Source: Neurosurg Rev. 2025 Aug 14;48(1):600. doi: 10.1007/s10143-025-03743-3 (PMC12350427; doi:10.1007/s10143-025-03743-3)
Supplement: Supplementary file 1 — (DOCX 188 KB) [file 10143_2025_3743_MOESM1_ESM.docx]

**Middle Meningeal Artery Embolization for Chronic Subdural Hematoma in the Very Elderly: An Individual Patient Pooled Meta-Analysis**

**Supplementary Material:**

**Supplementary Material, Figure 1:** Sankey diagrams showing the distribution between vascular access route and anesthesia type in elderly patients undergoing MMAE. (A) Patients aged 80–89 years. (B) Patients aged ≥90 years

**
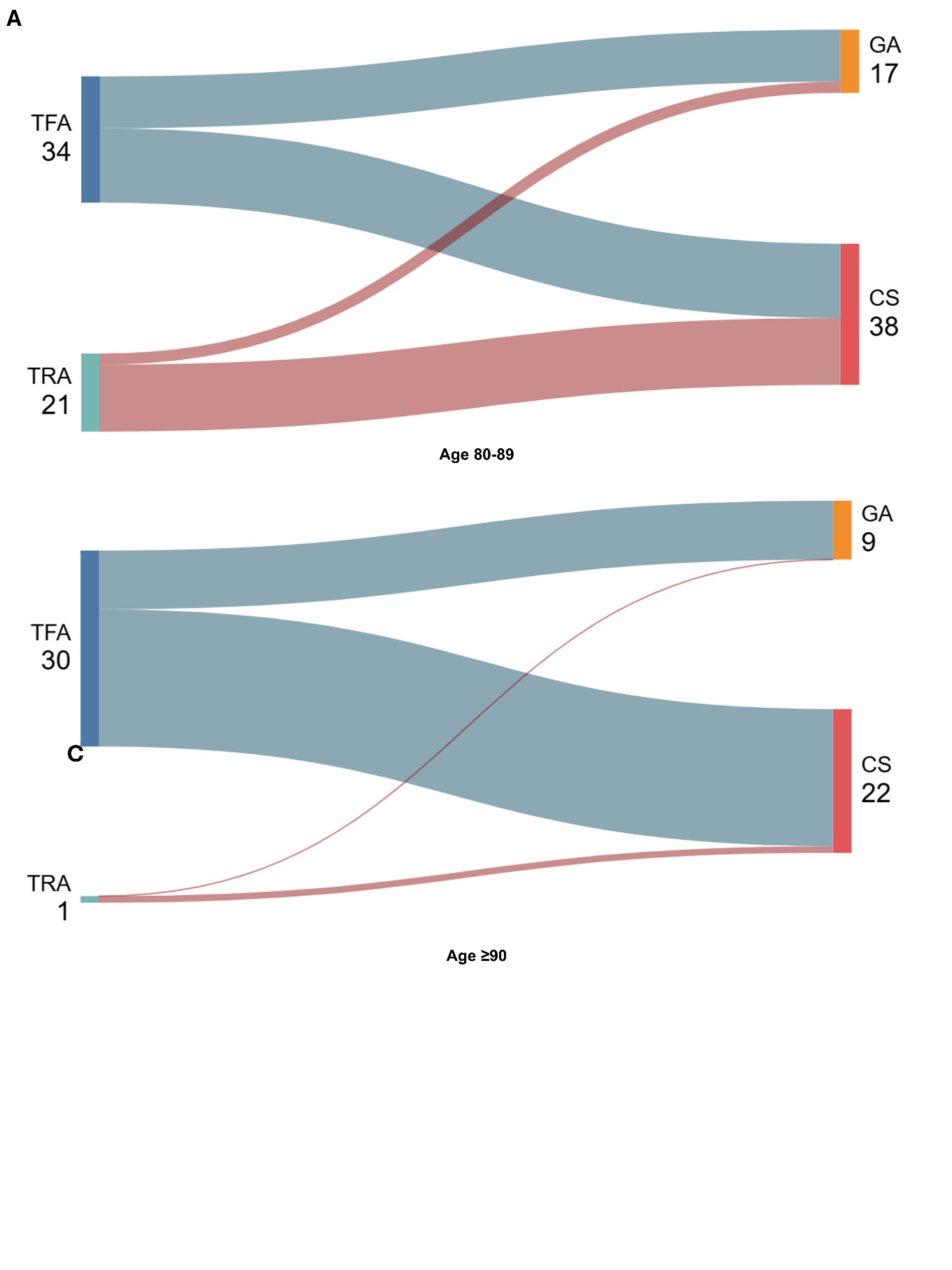
**

**Supplementary Material, Table 1:** List of the studies curated in the systemic review

| **Study** | **Publication Year** | **Journal** | **Study Design** | **SDH size (mm)** | **Total Sample size** | **Patients Aged ≥80 Years** | **Patients Aged 80–89 Years** | **Patients Aged ≥90 Years** |
| --- | --- | --- | --- | --- | --- | --- | --- | --- |
| Granstein et al 2025^1^ | 2025 | Clinical Neurology and Neurosurgery | Case series | Available | 21 | 21 | 0 | 21 |
| Kadono et al 2024^2^ | 2024 | Radiol Case Rep | Case report | NA | 2 | 1 | 1 | 0 |
| Seok et al 2023^3^ | 2023 | J. Cerebrovasc. Endovasc. Neurosurg. | Case Series | Available for each patient in the manuscript | 9 | 6 | 6 | 0 |
| Ferber et al 2023^4^ | 2023 | Brain Inj. | Case report | 16 | 1 | 1 | 1 | 0 |
| Cardoso et al 2023^5^ | 2023 | Am. J. Case Rep. | Case report | 13 | 1 | 1 | 0 | 1 |
| Wong et al2023^6^ | 2023 | Brain. Hemorrhages. | Case series | Available for each patient in the manuscript | 7 | 3 | 0 | 3 |
| Tanoue et al 2023^7^ | 2023 | World Neurosurg. | Case series | They provided volume instead of thickness | 15 | 6 | 5 | 1 |
| Dzaye et al 2023^8^ | 2023 | Daign. Intervent. Imaging | Case series | Available but not per patient | 11 | 3 | 3 | 0 |
| Saal-Zapata et al 2023^9^ | 2023 | J. Neurosci. Rural Pract. | Case series | Available for each patient in the manuscript | 5 | 5 | 3 | 2 |
| Dofuku et al 2023^10^ | 2023 | Neurol. Med.-Chir. | case series | Available but not per patient | 9 | 7 | 4 | 3 |
| Sarma et al 2022^11^ | 2022 | J. Neurosci. Rural Pract. | Case series | Could not access | 4 | 2 | 2 | 0 |
| Stanishevskiy et al 2022^12^ | 2022 | Surg. Neurol. Intl. | Case series | NA | 2 | 1 | 1 | 0 |
| Imai et al 2022^13^ | 2022 | Surg. Neurol. Intl. | Case report | NA | 1 | 1 | 1 | 0 |
| Nomura et al 2022^14^ | 2022 | Surg. Neurol. Intl. | Case series | NA | 4 | 2 | 2 | 0 |
| Entezami et al 2021^15^ | 2021 | Intervent. Neuroradiol. | case series | NA | 6 | 1 | 1 | 0 |
| Petrov et al 2021^16^ | 2021 | J. Clin. Med. | Case series | They provided volume instead of thickness | 10 | 2 | 2 | 0 |
| Sattur et al 2020^17^ | 2020 | World Neurosurg. | Case report | NA | 1 | 1 | 1 | 0 |
| Yajima et al 2020^18^ | 2020 | Clin. Neurol. Neurosurg. | case series | NA | 18 | 8 | 5 | 3 |
| Piergallini et al 2019^19^ | 2019 | World Neurosurg. | Case series | 37 (one one) | 2 | 2 | 2 | 0 |
| Entezami et al 2019^20^ | 2019 | Intervent. Neuroradiol. | case report | NA | 2 | 1 | 1 | 0 |
| Okuma et al 2019^21^ | 2019 | World Neurosurg. | case series | NA | 17 | 8 | 8 | 0 |
| Entezami et al 2019^22^ | 2019 | Headache | case report | 9 | 1 | 1 | 1 | 0 |
| Link et al 2018^23^ | 2018 | World Neurosurg. | Case series | Available for each patient in the manuscript | 6 | 1 | 1 | 0 |
| Mewada et al 2016^24^ | 2016 | World Neurosurg. | Case report | 20 | 1 | 1 | 1 | 0 |
| Tempaku et al 2015^25^ | 2015 | Intervent. Neuroradiol. | Case series | NA | 5 | 3 | 2 | 1 |
| THashimoto et al 2013^26^ | 2013 | Surg Neurol Int | Case series | NA | 5 | 1 | 1 | 0 |
| Hirai et al 2004^27^ | 2004 | Intervent. Neuroradiol. | Case series | NA | 2 | 1 | 1 | 0 |
| **Total Pooled cases** | | | |  | 168 | 91 | 56 | 35 |
| **Total cases included in final analysis** | | | |  | - | 86 | 55 | 31 |

**Reference to the studies curated in the systemic review**

1. Granstein JH, Fodor TB, Young M, et al. Middle meningeal artery embolization for chronic subdural hematoma in the nonagenarian population. *Clin Neurol Neurosurg*. 2025;249:108747. doi:10.1016/j.clineuro.2025.108747

2. Kadono Y, Kajikawa R, Tsuzuki T, Kishima H. Middle meningeal artery embolization for organized chronic subdural hematoma combined with minimal evacuation surgery: Two case reports. *Radiol Case Rep*. 2024;19(12):6328-6332. doi:10.1016/j.radcr.2024.09.062

3. Seok JH, Kim JH, Kwon TH, Byun J, Yoon WK. Middle meningeal artery embolization for chronic subdural hematoma in elderly patients at high risk of surgical treatment. *J Cerebrovasc Endovasc Neurosurg*. 2023;25(1):28-35. doi:10.7461/jcen.2022.E2022.08.003

4. Ferber A, Zhou ,Yi, and Greenwald B. Persistent facial nerve palsy after middle meningeal artery embolization for subdural hematoma: a case report. *Brain Inj*. 2023;37(5):457-460. doi:10.1080/02699052.2023.2166116

5. Cardoso ER. Single Pterional Burr Hole Coupled with Coagulation of the Middle Meningeal Artery for Management of a Chronic Subdural Hematoma in a 98-Year-Old Patient: Illustrative Case. *Am J Case Rep*. 2023;24:e940045-1-e940045-5. doi:10.12659/AJCR.940045

6. Wong GK, Cheung EY, Ng RY, Yu SC, Chan DY, Zhuang JT. Middle meningeal embolization for chronic subdural Hematoma: A case series of 7 patients and review of time course of resolution. *Brain Hemorrhages*. 2023;4(1):30-33. doi:10.1016/j.hest.2022.04.004

7. Tanoue S, Ono K, Toyooka T, Okawa H, Wada K, Shirotani T. The Short-Term Outcome of Middle Meningeal Artery Embolization for Chronic Subdural Hematoma with Mild Symptom: Case Series. *World Neurosurg*. 2023;171:e120-e125. doi:10.1016/j.wneu.2022.11.090

8. Dzaye O, Brahmbhatt A, Abajian A, et al. Middle meningeal artery embolization using cone-beam computed tomography augmented guidance in patients with cancer. *Diagn Interv Imaging*. 2023;104(7):368-372. doi:10.1016/j.diii.2023.03.007

9. Saal-Zapata G, Murga-Villanueva A, Walker M, Ghodke B, Rodríguez-Varela R. Safety and radiologic clearance of chronic subdural hematoma after endovascular embolization using SQUID 18 in patients older than 80 years. *J Neurosci Rural Pract*. 2023;14(2):336-341. doi:10.25259/JNRP_30_2022

10. DOFUKU S, SATO D, NAKAMURA R, et al. Sequential Middle Meningeal Artery Embolization after Burr Hole Surgery for Recurrent Chronic Subdural Hematoma. *Neurol Med Chir (Tokyo)*. 2022;63(1):17-22. doi:10.2176/jns-nmc.2022-0164

11. Sarma P, Garg M, Prem P, Gupta R. Embolization of the Middle Meningeal Artery for the Treatment of Chronic Subdural Hematoma: A Path Less Travelled So Far. *J Neurosci Rural Pract*. 2022;13(3):471-475. doi:10.1055/s-0042-1750704

12. Stanishevskiy A, Jakovenko A, Ryzhova M, et al. Microstructure of embolized capsule of chronic subdural hematoma. *Surg Neurol Int*. 2022;13:531. doi:10.25259/SNI_691_2022

13. Imai R, Akiyama T, Mizutani K, Toda M. A case of refractory chronic subdural hematoma and internal carotid artery stenosis sequentially treated with surgical drainage, middle meningeal artery embolization, and carotid artery stenting. *Surg Neurol Int*. 2022;13:342. doi:10.25259/SNI_505_2022

14. Nomura S, Haji K, Fujiyama Y, Nishimoto T, Oka F, Ishihara H. Endoscopically observed outer membrane of chronic subdural hematoma after endovascular embolization of middle meningeal artery. *Surg Neurol Int*. 2022;13:516. doi:10.25259/SNI_886_2022

15. Entezami P, Field NC, Dalfino JC. Outpatient management of chronic expanding subdural hematomas with endovascular embolization to minimize inpatient admissions during the COVID-19 viral pandemic. *Interv Neuroradiol*. 2021;27(5):716-721. doi:10.1177/1591019921996510

16. Petrov A, Ivanov A, Rozhchenko L, et al. Endovascular Treatment of Chronic Subdural Hematomas through Embolization: A Pilot Study with a Non-Adhesive Liquid Embolic Agent of Minimal Viscosity (Squid). *J Clin Med*. 2021;10(19):4436. doi:10.3390/jcm10194436

17. Sattur MG, Spiotta AM. Anomalous “Middle” Meningeal Artery from Basilar Artery and Implications for Neuroendovascular Surgery: Case Report and Review of Literature. *World Neurosurg*. 2020;133:84-89. doi:10.1016/j.wneu.2019.09.130

18. Yajima H, Kanaya H, Ogino M, Ueki K, Kim P. Middle meningeal artery embolization for chronic subdural hematoma with high risk of recurrence: A single institution experience. *Clin Neurol Neurosurg*. 2020;197:106097. doi:10.1016/j.clineuro.2020.106097

19. Piergallini L, Dargazanli C, Derraz I, Costalat V. Immediate Development of Dural Arteriovenous Fistula After Middle Meningeal Artery Embolization: First Angiographic Demonstration. *World Neurosurg*. 2019;128:606-610.e1. doi:10.1016/j.wneu.2019.04.246

20. Entezami P, Boulos A, Paul A, Nourollahzadeh E, Dalfino J. Contrast enhancement of chronic subdural hematomas after embolization of the middle meningeal artery. *Interv Neuroradiol*. 2019;25(5):596-600. doi:10.1177/1591019919843354

21. Okuma Y, Hirotsune N, Sato Y, Tanabe T, Muraoka K, Nishino S. Midterm Follow-Up of Patients with Middle Meningeal Artery Embolization in Intractable Chronic Subdural Hematoma. *World Neurosurg*. 2019;126:e671-e678. doi:10.1016/j.wneu.2019.02.121

22. Entezami P, Nourollahzadeh E, Dalfino J. Embolization of Middle Meningeal Artery for the Treatment of Headaches Induced by Chronic Subdural Hematoma: A Case Report. *Headache J Head Face Pain*. 2019;59(4):615-618. doi:10.1111/head.13519

23. Link TW, Schwarz JT, Paine SM, Kamel H, Knopman J. Middle Meningeal Artery Embolization for Recurrent Chronic Subdural Hematoma: A Case Series. *World Neurosurg*. 2018;118:e570-e574. doi:10.1016/j.wneu.2018.06.241

24. Mewada T, Ohshima T, Yamamoto T, Goto S, Kato Y. Usefulness of Embolization for Iatrogenic Dural Arteriovenous Fistula Associated with Recurrent Chronic Subdural Hematoma: A Case Report and Literature Review. *World Neurosurg*. 2016;92:584.e7-584.e10. doi:10.1016/j.wneu.2016.05.042

25. Tempaku A, Yamauchi S, Ikeda H, et al. Usefulness of interventional embolization of the middle meningeal artery for recurrent chronic subdural hematoma: Five cases and a review of the literature. *Interv Neuroradiol*. 2015;21(3):366-371. doi:10.1177/1591019915583224

26. Hashimoto T, Ohashi T, Watanabe D, et al. Usefulness of embolization of the middle meningeal artery for refractory chronic subdural hematomas. *Surg Neurol Int*. 2013;4:104. doi:10.4103/2152-7806.116679

27. Hirai S, Ono J, Odaki M, Serizawa T, Nagano O. Embolization of the Middle Meningeal Artery for Refractory Chronic Subdural Haematoma: Usefulness for Patients under Anticoagulant Therapy. *Interv Neuroradiol*. 2004;10(2_suppl):101-104. doi:10.1177/15910199040100S218
